# Cellular and Molecular Mechanisms of Neuroprotection in Deep Brain Stimulation for Parkinson’s Disease

**DOI:** 10.3390/biomedicines14010019

**Published:** 2025-12-21

**Authors:** Aiswarya Nag, Siddharth Shah, Brandon Lucke-Wold

**Affiliations:** 1 Department of Neurosurgery, University of Florida, Gainesville, FL 32608, USA; aish.nag97@gmail.com (A.N.); brandon.lucke-wold@neurosurgery.ufl.edu (B.L.-W.); 2 Department of Neurosurgery, University of Maryland School of Medicine, Baltimore, MD 20201, USA

**Keywords:** deep brain stimulation, Parkinson’s disease, neuroprotection, neurotrophic factors, synaptic plasticity, neuroinflammation, oxidative stress, disease modification

## Abstract

Deep brain stimulation (DBS) is an established therapy for motor symptom management in Parkinson’s disease (PD), yet emerging evidence suggests that its effects may extend beyond functional circuit modulation to include cellular and molecular mechanisms with potential neuroprotective significance. This review synthesizes current evidence on the neuroprotective mechanisms of DBS, with an emphasis on preclinical and clinical studies that highlight its effects on neuronal survival, trophic support, oxidative stress, inflammation, synaptic plasticity, and network homeostasis. Preclinical data indicate that DBS reduces dopaminergic neuron degeneration, enhances brain-derived neurotrophic factor (BDNF) signaling, preserves mitochondrial function, attenuates neuroinflammation, and fosters synaptic remodeling. Clinical studies provide convergent, though less definitive, evidence from imaging, fluid biomarkers, and long-term outcomes supporting potential disease-modifying effects. These findings underscore a shift in the conceptualization of DBS from purely symptomatic relief toward modulation of underlying pathogenic processes. DBS holds promise as a neuroprotective therapy for PD, but critical gaps remain in validating these mechanisms in patients. Future directions include the development of biomarker-driven longitudinal studies, refinement of adaptive stimulation strategies, integration with adjunctive disease-modifying strategies, and exploration of personalized approaches based on molecular and network signatures. By bridging mechanistic understanding with translational innovation, DBS may evolve into a precision therapy capable of altering the progression trajectory of PD.

## 1. Introduction

**Figure 1:**
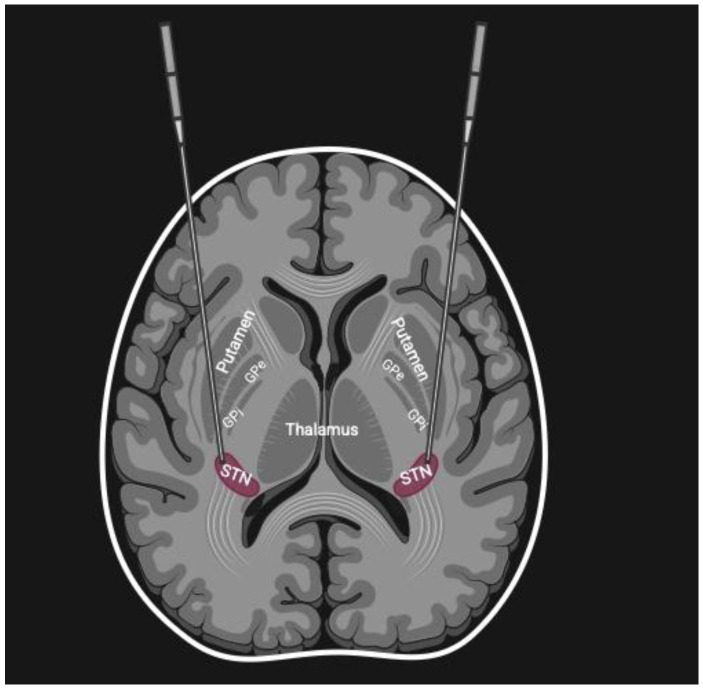
Illustrates concept of subthalamic deep brain stimulation (DBS).

Parkinson’s disease (PD) is a progressive neurodegenerative disorder defined clinically by selective degeneration of dopaminergic neurons of the substantia nigra pars compacta and resulting striatal dopamine loss. Parkinson’s disease pathology extends beyond dopamine insufficiency and encompasses mitochondrial dysfunction, disrupted protein homeostasis, oxidative stress, neuroinflammation, and maladaptive basal ganglia–thalamocortical circuitry modifications. All these cellular and molecular defects work synergistically and contribute to the progression of both motor and non-motor features of the illness [1,2]. Deep brain stimulation (DBS) of targets such as the subthalamic nucleus (STN) and globus pallidus internus (GPi) is an established therapy of end-stage PD [Figure 1] and produces substantial and sustained improvement of motor and quality-of-life symptoms [2]. STN-DBS modulates brain circuit dynamics, enhancing alpha- and gamma-like neuronal firing, restoring thalamo-cortical signaling, and facilitating basal ganglia–mediated ‘OFF’-to ‘ON’ transitions [3]. However, emerging evidence indicates that DBS is not merely a “neuromodulatory switch” for controlling motor symptoms. Long-term DBS appears to gradually reshape neuronal circuits, promoting synaptic plasticity and even neurogenesis [2,4]. Figure 1 demonstrates the concept of subthalamic deep brain stimulation electrodes placement.

Deep brain stimulation (DBS) systems traditionally operate in an open-loop manner: an implantable neurostimulator with an internal battery delivers continuous electrical stimulation at fixed, clinician-programmed settings. The vast majority of currently implanted devices do not acquire neuronal or physiological signals, and stimulation adjustments require manual reprogramming. Only recently have sensing-enabled platforms been introduced. The Medtronic Percept PC, commercially available for approximately one year, incorporates an onboard low-noise amplifier capable of recording local field potentials (LFPs) from the implanted electrodes. This functionality permits chronic neural sensing and supports the development of adaptive or closed-loop stimulation paradigms, but is not representative of most DBS systems in clinical use. It is typically implanted under the skin in the chest or abdomen, modulating neurons near the electrode tips to relieve disease symptoms [5]. DBS delivers continuous, programmable electrical stimulation to targeted brain structures via chronically implanted electrodes connected to an internalized stimulator. The stimulator can be periodically tuned to adapt therapy to the patient’s evolving symptoms [6]. The original “inhibition hypothesis” suggested that DBS works by suppressing overactive STN or GPi neurons, thereby restoring thalamic activity like the effects of a lesion. However, studies correcting for stimulation artifacts have shown that DBS can increase STN activity during stimulation, challenging this traditional model. Moreover, although STN and GPi stimulation share many overlapping clinical effects, target selection also depends on factors such as medication reduction and differential cognitive outcomes [7]. It exerts far broader effects at cellular, molecular, and network levels, modulating dysfunctional circuits (“circuitopathies”) toward more physiological states [8]. DBS targets are selected according to symptom profile: STN stimulation can reduce levodopa requirements, while GPi stimulation may alleviate dyskinesia and psychiatric symptoms. Overall, both approaches similarly improve motor function and daily living in PD [9].

DBS has revolutionized Parkinson’s disease management and shows promising neuroprotective potential across neurodegenerative and neuroinflammatory disorders like Alzheimer’s disease and epilepsy. In Parkinson’s models, chronic STN-DBS preserves dopaminergic neurons and improves motor function through enhanced neurotrophic signaling, synaptic remodeling, and anti-apoptotic mechanisms. In Alzheimer’s disease, DBS of memory circuits such as the fornix and nucleus basalis of Meynert promotes hippocampal neurogenesis, synaptic plasticity, and clearance of tau and amyloid, suggesting cognitive stabilization in early cases. Similarly, anterior thalamic nucleus DBS in epilepsy reduces seizure frequency, neuronal loss, and inflammatory injury, underscoring its potential neuroprotective role across diverse disease contexts. Preclinical studies have verified that DBS can suppress dopaminergic neuronal loss and reduce excitotoxicity arising from overactive glutamatergic projections, which originate in the STN. Beyond reducing excitotoxicity, STN-DBS may support neuronal survival by promoting the release of neurotrophic factors [2]. DBS directly modulates glial activity, augments trophic support, and affects relevant intracellular signaling cascades [7].

Clinical data suggest that biochemical biomarkers from cerebrospinal fluid, peripheral body fluids, and tissue samples, as well as imaging biomarkers such as SPECT, PET, and MRI, can help characterize disease progression in PD. While these biomarkers do not directly “guide” DBS programming, they may inform patient selection, timing of surgery, and the assessment of disease state or treatment response in individuals undergoing DBS [10]. DBS is proposed to have disease-modifying effects beyond its strong symptomatic benefits. These include reducing excitotoxic input from STN glutamatergic projections to the substantia nigra pars compacta (SNc), enhancing brain-derived neurotrophic factor (BDNF) signaling via the tropomyosin receptor kinase B (TrkB) receptor in SNc neurons, and directly mitigating α-synuclein toxicity [11].

DBS is now classified as a form of neuromodulation, altering neural function through controlled electrical stimulation. Its effects are largely immediate, reversible, adjustable, and titratable without causing permanent neural damage. Unlike lesioning techniques, DBS can be applied bilaterally during a single surgical session without typically inducing severe side effects. Although bilateral lesions are also possible, they are generally performed in a staged manner due to the higher risk of cumulative adverse effects, an important distinction when comparing the safety profiles of the two approaches [8]. Although DBS is clinically effective in PD and target selection is guided by patient-specific features, corresponding cellular and molecular mechanisms of potential neuroprotective activities remain largely unclear [4]. This review sets out to examine emerging cellular and molecular evidence for DBS-mediated neuroprotection in Parkinson’s disease, framing DBS as both a circuit-level modulator and a regulator of neuronal and glial homeostasis, with implications for disease-modifying strategies and future therapeutic development.

## 2. Pathophysiological Landscape of Parkinson’s Disease

The neuropathological hallmark of Parkinson’s disease (PD) is the progressive degeneration of dopaminergic neurons within the substantia nigra pars compacta (SNc) projecting to the striatum via the nigrostriatal pathway, resulting in striatal dopamine depletion and disrupted modulation of the basal ganglia–thalamocortical circuitry. This degeneration underlies the cardinal motor features of PD, including resting tremor, rigidity, bradykinesia, postural instability, and the characteristic Parkinsonian gait. On a microscopic level, Parkinson’s disease (PD) is characterized by the presence of Lewy bodies which are cytoplasmic inclusions composed primarily of misfolded α-synuclein, ubiquitin, and synphilin-1 which reflect abnormal protein aggregation and impaired proteostasis. Although Lewy bodies are a common pathological hallmark, they are not universal in all clinically diagnosed cases of PD. Lewy pathology often appears initially in peripheral or lower brainstem regions before progressing rostrally to cortical areas, supporting a prion-like model of α-synuclein propagation. Variability in the distribution and burden of α-synuclein pathology contributes to the heterogeneity of PD phenotypes and progression rates, but this variation remains within the spectrum of PD itself. Disorders such as PSP and corticobasal degeneration represent distinct tauopathies rather than subtypes of PD and are therefore not discussed here [12].

PSP comprises two major subtypes—PSP-Richardson’s syndrome (PSP-RS) and PSP-parkinsonism (PSP-P)—which differ in symptom profile, rate of progression, and regional tau burden. Recent work by Alster et al. (2024) [13] has identified compensatory upregulation of neurotrophic pathways, including glial-derived neurotrophic factor (GDNF), in these PSP subtypes, suggesting that neurotrophic alterations may represent an endogenous response to tau-mediated neuronal injury. Notably, PSP-P appears to exhibit stronger GDNF-related compensatory activity than PSP-RS, potentially contributing to its comparatively slower clinical progression. These findings highlight that neurotrophic signaling is not uniformly depleted across atypical parkinsonian disorders but may vary by subtype, with implications for understanding selective vulnerability and therapeutic modulation [13].

**Figure 2:**
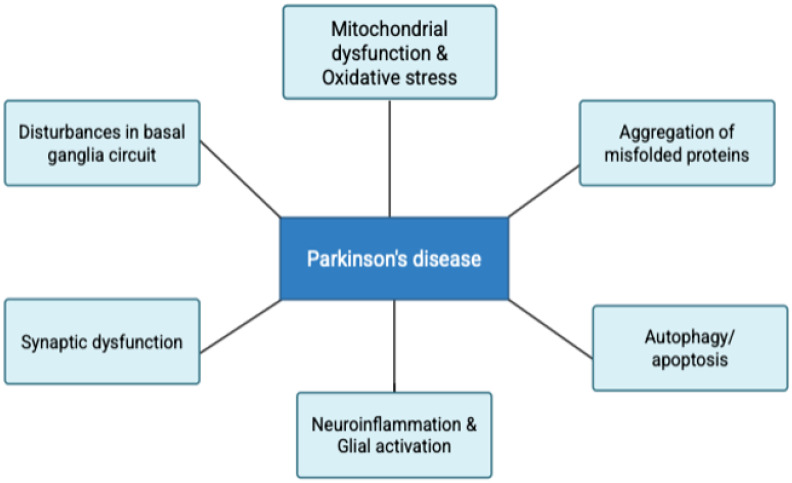
Diagram showing pathophysiological process involved in Parkinson’s disease.

At the cellular level, multiple converging mechanisms contribute to dopaminergic neurodegeneration—oxidative stress, mitochondrial dysfunction, endoplasmic reticulum stress, aberrant protein folding, excitotoxicity, neuroinflammation, apoptosis, and the depletion of neurotrophic factors such as GDNF and neurturin [Figure 2]. Recent findings also highlight that PD pathology may originate in axonal terminals rather than neuronal somata, emphasizing early synaptic dysfunction in disease onset. Importantly, neuroinflammation—mediated by activated microglia and astrocytes—may act both as a causative driver of neuronal injury and as a secondary response to accumulating cellular damage, forming a self-perpetuating cycle of neurodegeneration. Moreover, systemic factors such as aging and environmental toxins can amplify these processes, overwhelming compensatory repair mechanisms [12].

In recent years, epigenetic mechanisms—including DNA methylation, chromatin remodeling, and gene regulation by non-coding RNAs—have emerged as potential contributors to PD pathogenesis. These modifications, influenced by genetic background, environmental exposures, and lifestyle factors from prenatal stages onward, may represent the missing link connecting PD risk factors to disease development. Thus, Parkinson’s disease is now recognized as a multifactorial and multisystem disorder—one in which interplay among oxidative, inflammatory, and proteostatic pathways shapes both disease onset and the course of distinct PD subtypes [12,14]. Figure 2 shows the pathophysiological process involved in PD.

### 2.1. Mitochondrial Function and Oxidative Stress

Mitochondrial dysfunction and oxidative stress play a central role in the pathogenesis of PD. Mitochondrial dysfunction plays a central role in Parkinson’s disease pathogenesis, with mtDNA mutations and complex I inhibition leading to dopaminergic neuron loss. Evidence from toxin models like MPTP (a neurotoxin that induces Parkinsonian syndromes across species by inhibiting mitochondrial complex I) shows reduced complex I activity in PD patients strongly support mitochondria as key contributors to neuronal degeneration [15]. Impairments of oxidative phosphorylation and lowered complex I activity lead to dysfunctional ATP synthesis and increased ROS emission. Oxidative stress contributes to lipid peroxidation, protein misfolding, DNA damage, and activation of apoptotic pathways [16]. The most effective current treatment for Parkinson’s disease remains dopaminergic replacement therapy, primarily through levodopa, which replenishes dopamine to improve motor symptoms. However, long-term use leads to complications such as motor fluctuations and dyskinesias. These complications are driven largely by maladaptive plasticity within basal ganglia circuits, including pulsatile dopaminergic stimulation, altered receptor trafficking, and abnormal downstream signaling, rather than by oxidative dopamine metabolism alone. Although mechanisms related to oxidative stress such as reduced glutathione and elevated homocysteine may contribute to overall neurodegenerative processes, they are not the primary drivers of levodopa-induced motor complications [17]. Antioxidants like vitamin E, vitamin C, creatine, CoQ10 offer limited yet crucial therapeutic potential by inducing protective proteins and neutralizing ROS [15,16]. Over the past decade, mitochondria-targeted antioxidants (MTAs) have advanced through conjugation with lipophilic cations like triphenylphosphonium (TPP), enabling selective accumulation within mitochondria driven by membrane potentials. Compounds such as MitoQ, MitoVitE, and MitoTEMPOL demonstrate enhanced mitochondrial uptake and protection against oxidative damage, showing promise for neurodegenerative diseases [15].

### 2.2. Protein Misfolding and α-Synuclein

The abnormal accumulation and oligomerization of the protein alpha-synuclein (α-syn) into Lewy bodies and Lewy neurites contribute to the loss of dopaminergic neurons in the substantia nigra in Parkinson’s disease, making α-syn a promising therapeutic target for disease-modifying interventions [18,19]. Aggregated and misfolded α-syn disrupts synaptic vesicle release and trafficking, impairs synaptic transmission, compromises mitochondrial integrity, and spreads in a prion-like manner throughout neuronal circuits. Impaired protein clearance mechanisms, such as autophagy and proteasomal degradation, further promote α-syn accumulation and the resulting toxicity [19]. The first evidence for α-synuclein oligomers in PD came from their detection in post-mortem brain samples. More recent studies have also identified these oligomers in the plasma and cerebrospinal fluid of PD patients [20]. Acute DBS of the SNc decreases the accumulation and abnormal aggregation of α-synuclein in both cultured neurons (in vitro) and the substantia nigra (in vivo). This suggests that DBS may extend beyond symptom relief, offering potential disease-modifying effects by targeting pathological protein accumulation in neurodegenerative disorders. Using proteomics and Western blotting, CSF protein changes in PD patients before and after DBS was identified which revealed alterations in several CSF proteins during the perioperative and follow-up periods. These changes may result from direct biophysical effects or activation of cellular degradation pathways, offering a novel approach to restore cellular homeostasis in Parkinson’s disease [18,21].

### 2.3. Neuroinflammation and Glial Activation

Neuroinflammation is a key feature of PD and plays a central role in its progression. Microglia are the brain’s primary immune cells, normally maintaining homeostasis and surveilling for damage. In PD, they respond to α-synuclein aggregates, apoptotic cells, and other pathological signals via pattern recognition receptors, initially promoting neuronal survival. Activated microglia can adopt a range of phenotypes, commonly simplified as proinflammatory M1 or anti-inflammatory M2 states. M1 microglia release cytokines, ROS, and neurotoxic molecules, promoting apoptosis and dopaminergic neuron degeneration, while M2 microglia secrete trophic factors that support repair and homeostasis. In Parkinson’s disease, M1 activation—triggered by DAMPs, misfolded α-synuclein, and astrocyte signals—contributes to neuronal loss and disease progression. However, chronic or excessive activation leads to sustained release of proinflammatory cytokines (e.g., TNF-α, IL-1β, IL-6), contributing to neurodegeneration. PD-associated gene mutations (SNCA, LRRK2, GBA, PINK1, Parkin) can impair microglial functions such as autophagy, phagocytosis, and mitochondrial activity, exacerbating neuroinflammation and dopaminergic neuron loss. Altered microglial gene signatures and cytokine profiles in both brain and CSF suggest their central role in disease progression and potential as biomarkers [22,23]. The use of broad-spectrum steroidal and non-steroidal anti-inflammatory drugs (NSAIDs), specific microglial inhibitors, and anti-inflammatory cytokines has helped clarify the role of microglial activation in Parkinson’s disease–related neuroinflammation. These studies also suggest that targeting the specific processes underlying microglial activation could offer a potential therapeutic strategy for PD [23].

### 2.4. Synaptic Dysfunction and Circuit Remodeling

The hallmark motor symptoms of PD arise from disrupted cortico-striatal circuitry due to progressive nigrostriatal dopaminergic degeneration [24]. Recent research has highlighted that α-syn induced synaptic dysfunction represents one of the earliest pathological events in PD, preceding Lewy pathology and dopaminergic neuron loss. At the presynaptic level, α-syn aggregation disrupts axonal microtubule transport, impairing both anterograde delivery of synaptic components and retrograde signaling essential for neuronal survival and neurite growth. These aggregates progressively compromise dopamine (DA) release machinery, potentially through interactions with oxidized DA that promote further α-syn accumulation. Postsynaptically, α-syn also alters synaptic plasticity within the striatum. Given dopamine’s role in modulating striatal neuron activity, early α-syn–induced changes in postsynaptic signaling may precede overt DA depletion [25]. These changes within circuits not only trigger motor impairment but are also funneled back into cellular stress cascades. Consequently, altered resting-state functional connectivity in cortical and brainstem networks has emerged as both a diagnostic and progression marker for PD [24].

Viewed collectively, PD emerge from a web of cellular and molecular insults—mitochondrial dysfunction, oxidative stress, protein aggregation, neuroinflammation, maladaptive circuit reorganization, misfolded proteins, and genetic mutations, which progressively compromise neuronal integrity [26]. This multifactorial framework raises the question of how DBS may confer neuroprotection—not only by modulating neural network dynamics but also by influencing the molecular and cellular pathways underlying neurodegeneration.

## 3. DBS Beyond Circuit Modulation: Evidence of Neuroprotection

**Figure 3:**
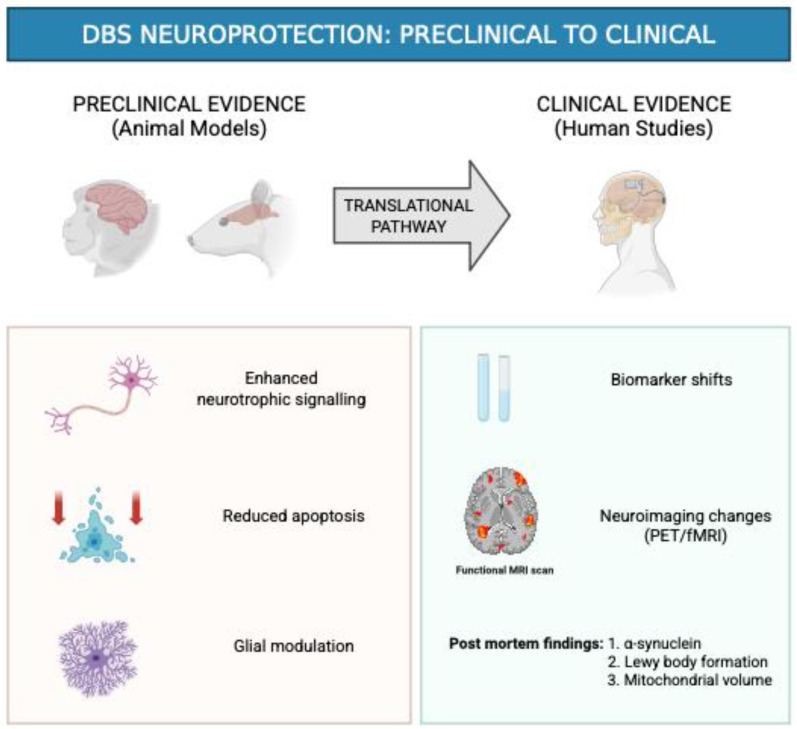
DBS neuroprotection effects in Preclinical & Clinical studies.

Subthalamic nucleus deep brain stimulation (STN-DBS) has shown potential to slow the progression of PD. STN-DBS was shown to enhance mitophagy in dopaminergic neurons through an mTOR-dependent pathway, which reduced oxidative stress by removing damaged mitochondria. This process also decreased the release of apoptosis-inducing factors from mitochondria, ultimately contributing to the neuroprotective effects of STN-DBS on dopaminergic neurons in the substantia nigra in PD [Figure 3] [27]. High-frequency stimulation (HFS) of the subthalamic nucleus (>50–180 Hz) can mimic the effects of ablative lesions, producing functional inhibition in neuronal nuclei while exciting fiber bundles. In PD, this frequency-dependent effect underlies the therapeutic and potential neuroprotective benefits of STN-DBS by reversibly modulating targeted brain regions [28,29]. The reversible nature of stimulation permits periodic assessment of the underlying disease state during treatment, an evaluation not possible with irreversible lesioning techniques [28]. Clinical improvements in tremor, rigidity, and bradykinesia are believed to arise from the suppression of pathological oscillations and the restoration of basal ganglia thalamocortical network activity [12]. Nevertheless, cumulative evidence of preclinical, translational, and clinical work has suggested that DBS has the ability to do more than just network modulation and can excite cellular and molecular processes favoring neuronal survival, neurodegeneration inhibition, and shaping of PD evolution [8].

### 3.1. Preclinical Evidence of Neuroprotection

Animal models were central to uncovering potential neuroprotective profiles of DBS [27].

Rodent models: STN-DBS after 6-hydroxydopamine (6-OHDA)-induced lesions reduced dopamine neuron loss of the substantia nigra pars compacta (SNc), preserved striatal dopamine terminals and improved performance [29]. High-frequency STN-DBS protects SNc neurons in PD rats by reducing apoptosis and enhancing neuronal excitability. This neuroprotective effect appears linked to modulation of neurotransmitter distribution and metabolism, particularly involving GABAergic pathways [30]. DBS may also promote the expression of brain-derived neurotrophic factor (BDNF), an anterogradely transported neurotrophin that supports the survival of dopaminergic neurons. In an A53T α-synuclein PD rat model, STN-DBS not only improved motor performance but also increased survival of dopaminergic SN neurons, supporting its potential neuroprotective and disease-modifying effects beyond symptomatic relief [31].

MPTP mouse models: In the MPTP mouse model, motor deficits and dopaminergic neuron loss became pronounced around four weeks post-injection, with further decline through week six. STN-DBS was initiated at four weeks to target established PD-like pathology and functional impairment. The stimulation improved rotarod motor performance and mitigated dopaminergic neuron loss in the substantia nigra. Although TH+ neurites in the striatum increased slightly, the change was not statistically significant, indicating that the primary neuroprotective effect of STN-DBS was localized to substantia nigra dopaminergic neurons [27]. DBS reduced progressive nigral dopaminergic neuron loss and reduced apoptosis in the hippocampus and cortex, accompanied by downregulation of apoptosis-related proteins (caspase-3, caspase-8, and Bid). It also increased antioxidant and cholinergic enzyme activity (superoxide dismutase, glutathione peroxidase, choline acetyltransferase) while decreasing oxidative stress markers and acetylcholine esterase activity. Notably, DBS-treated rodents exhibited persistent locomotor activity improvements beyond those attributable to simple circuit compensation [32].

Nonhuman primates: Studies in non-human primates (NHPs) have been pivotal in understanding basal ganglia function and movement disorder pathophysiology. NHP models closely mimic human brains, allowing accurate evaluation of interventions like DBS. DBS and ablative procedures, informed by NHP research, effectively treat Parkinsonism and hyperkinetic disorders by suppressing disruptive basal ganglia output, highlighting the translational importance of NHP studies in developing therapies [33]. High frequency STN-DBS in MPTP-treated monkeys preserved dopamine transporter binding and striatal dopamine content relative to controls [34]. These preclinical studies remain crucial for understanding PD and refining neurosurgical therapies [35].

### 3.2. Clinical Observations Supporting Neuroprotection

Direct evidence of neuroprotection in humans remains elusive. Several lines of clinical, imaging, and postmortem data have been interpreted as suggestive of possible neuroprotective effects, but these findings must be distinguished from symptomatic effects that occur only during active stimulation. Neuroprotection, in contrast, would require persistent benefit or slowed degeneration even when stimulation is not present.

Neuroimaging studies: Longitudinal PET and SPECT studies have reported reduced rates of striatal dopamine transporter decline in DBS-treated patients compared with pharmacologically managed controls, findings that correlate with symptomatic improvement [36,37,38]. However, these imaging changes occur during active stimulation and likely reflect altered neural activity rather than structural preservation. For example, high-frequency STN-DBS improves motor symptoms and reduces levodopa dependence, and SPECT imaging demonstrates increased cerebral blood flow in the pre-supplementary and premotor cortices during stimulation, correlating with functional recovery [37]. These effects are therefore best considered physiological consequences of ongoing stimulation, not direct evidence of slowed disease progression. Resting-state fMRI in early PD has revealed reduced connectivity within basal ganglia networks, which may serve as a marker of early dysfunction. Although imaging modalities remain promising exploratory biomarkers in PD, they assess neural activity rather than enduring structural integrity, limiting their ability to demonstrate neuroprotection. Their cost, limited availability, and radiation exposure further constrain their use, making them more suitable as confirmatory rather than screening tools [10].

Biomarker shifts: A variety of electrophysiologic and biochemical biomarkers have been evaluated to understand DBS effects in PD, including changes in oscillatory activity, gene expression, neuropeptides, metabolic intermediates, and inflammatory markers [38]. Among these, local field potentials and beta-band activity remain the most clinically relevant biomarkers for monitoring stimulation efficacy, but they do not indicate long-term structural preservation. Biomarkers such as microglial activation markers, neurofilament proteins in CSF, extracellular dopamine and GABA, and metabolites including DOPAC and HVA measured via intracerebral microdialysis reflect acute changes in neural activity and neurotransmission during stimulation. These shifts do not persist once stimulation is stopped and therefore cannot be interpreted as neuroprotective. Although serum biomarkers may help monitor PD severity, inconsistencies between biomarker fluctuations and clinical outcomes currently limit their reliability [39]. Importantly, STN-DBS does not appear to accelerate neurodegeneration, but this alone does not constitute evidence of slowed progression. Distinguishing between symptomatic neural activation and true disease-modifying effects remains a central challenge.

Postmortem analyses: Postmortem studies provide structural insights but remain limited by their static nature. Analyses of PD brains consistently demonstrate α-synuclein and Lewy body pathology [38], but the ability to infer neuroprotection is extremely restricted. Mallach et al. (2019) [40] examined three controls, four PD cases, and only one PD case treated with STN-DBS. As expected, PD brains showed reduced mitochondrial volume and increased distance between mitochondria and presynaptic terminals, whereas the single DBS-treated brain exhibited mitochondrial volumes comparable to controls, suggesting a possible restoration of mitochondrial biomass [40]. Although intriguing, this observation is derived from a single patient and therefore cannot support any generalizable conclusions regarding neuroprotection. It remains unclear whether these differences reflect individual variability, sampling effects, or changes occurring only during chronic stimulation. Furthermore, post-mortem tissue cannot assess mitochondrial efficiency, biogenesis, turnover, or dynamic adaptation. Much larger cohorts and longitudinal in vivo studies are required to determine whether DBS produces any reproducible neuroprotective effect in humans.

Clinical course: Preclinical studies suggest that STN-DBS may preserve nigral dopaminergic neurons and enhance striatal dopamine levels in early PD models. Clinically, early use of STN-DBS yields substantial symptomatic benefits, including improved motor function, better quality of life, and reduced levodopa-related complications, with predominantly mild and transient side effects. Trials such as EARLYSTIM demonstrate these symptomatic advantages, particularly in younger or rapidly progressing patients [41]. However, these symptomatic improvements should not be interpreted as evidence of neuroprotection. Long-term human studies, including PET imaging and extended follow-ups, have not demonstrated consistent slowing of disease progression or modification of non-dopaminergic or late-stage symptoms. Imaging findings mainly reflect dopamine metabolism and neural activity during stimulation rather than preservation of neuronal structure. Clinical studies are further complicated by placebo effects, rater bias, and symptomatic confounding. Thus, while DBS provides robust and well-established symptomatic benefit, current human evidence does not confirm that DBS alters the underlying progression of Parkinson’s disease, and any potential disease-modifying effects remain speculative and unproven [42,43].

## 4. Cellular and Molecular Mechanisms of DBS-Induced Neuroprotection

Even though the symptomatic efficacy of DBS in Parkinson’s is evident, an emerging consensus proposes that its impact may extend within the cellular and molecular realm. DBS induces a constellation of mechanisms that collectively increase neuronal resilience, regulate glial response, and elicit neuroprotective signaling cascades. These mechanisms are apparently integrated, reflecting the complex dynamic between neuronal performance, glia activity, and molecular cascades [44].

**Figure 4:**
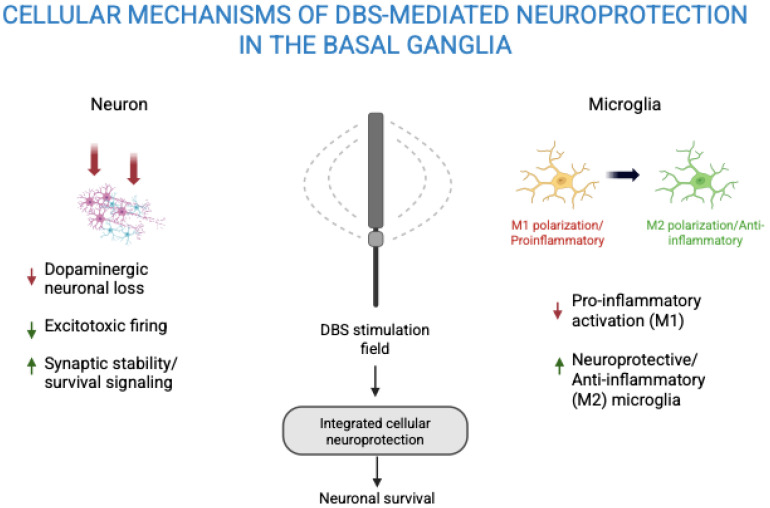
Cellular mechanisms of DBS induced neuroprotection.

**Figure 5:**
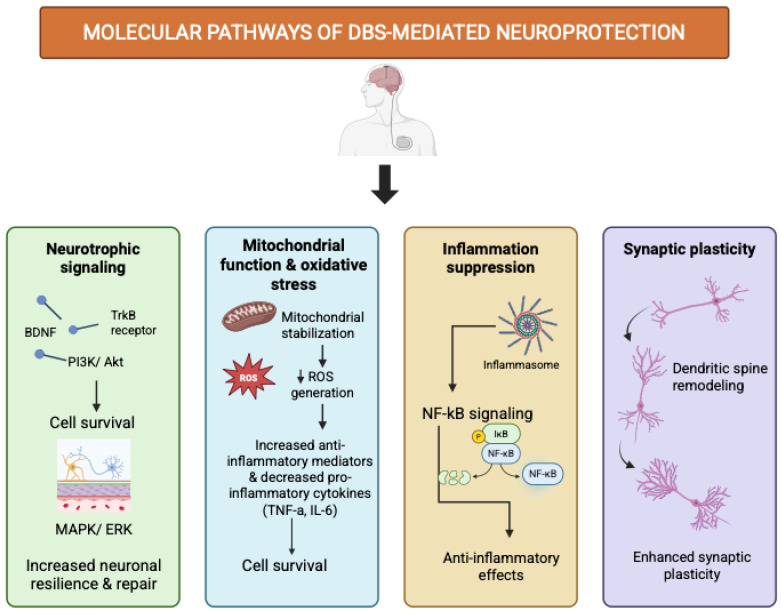
Molecular mechanisms of DBS induced neuroprotection.

Macroscopic electrical stimulation by STN-DBS likely triggers molecular and cellular signaling cascades through multilevel circuit interactions within the cortico–basal ganglia–thalamo–cortical network [Figure 4 and Figure 5]. Stefani A. et al., highlights that DBS effects extend beyond simple modulation of firing rates in the direct and indirect pathways, incorporating additional structures such as the hyperdirect pathway and pedunculopontine nucleus (PPN). High-frequency stimulation of the STN induces both orthodromic and antidromic activation, influencing upstream cortical neurons and downstream nuclei like the GPi and SNr. This bidirectional propagation alters excitatory (glutamatergic) and inhibitory (GABAergic) fluxes, leading to fast reorganization of oscillatory band frequencies and local nucleotide signaling (cAMP/cGMP). These neurochemical shifts can influence intracellular second messengers and transcriptional activity, potentially upregulating trophic factors such as BDNF. The recruitment of cholinergic and thalamostriatal pathways further suggests that DBS-induced changes in network activity can modulate neuromodulator release and microglial activation states, serving as biological intermediates between electrical input and molecular adaptation. In parallel, evidence from activation and disruption models suggests that DBS entrains neuronal firing and reorganizes pathological synchrony across the basal ganglia. By interrupting aberrant oscillations and restoring physiological firing patterns, DBS reduces maladaptive inhibitory drive from GPi to the thalamus and normalizes cortical excitability. This circuit-level rebalancing may secondarily activate intracellular signaling cascades via calcium influx and NMDA receptor–dependent mechanisms in cortical and nigral projections. Such activity-dependent signaling is known to stimulate BDNF–TrkB pathways, promoting synaptic plasticity and neuronal resilience. Moreover, antidromic activation of cortico-subthalamic afferents may contribute to retrograde trophic signaling reaching the substantia nigra compacta (SNc), linking altered firing dynamics to molecular neuroprotection. Thus, within this framework, DBS-induced molecular effects emerge not as isolated cellular phenomena but as downstream consequences of distributed electrophysiological reorganization across interconnected basal ganglia–cortical loops [3].

### 4.1. Neuronal Mechanisms

#### 4.1.1. Mitochondrial Stability and Oxidative Stress Reduction

Neurons rely on healthy mitochondria for energy, transport, and stress resilience, making mitochondrial dysfunction a key contributor to Parkinson’s disease through impaired bioenergetics, dynamics, transport, and quality control. Oxidative stress-driven mitochondrial dysfunction is central to PD pathogenesis. Neuroprotective effects are exerted by restoring mitochondrial biogenesis, normalizing mitophagy, and stabilizing autophagy pathways via PINK1/Parkin, AMPK–mTOR signaling, and BDNF modulation offering potential disease-modifying benefits beyond symptomatic relief. DBS has been shown both to enhance mitochondrial respiratory chain activity, reduce ROS production, and maintain mitochondrial membrane potential in primate and rodent PD models [15,45,46]. Several endogenous and natural compounds show mitochondrial-protective effects in Parkinson’s disease models by reducing ROS, preserving membrane potential, and preventing apoptosis. Prominent examples include melatonin, mitoQ, α-lipoic acid, ursodeoxycholic acid, resveratrol, quercetin all of which enhance antioxidant defenses and mitochondrial integrity combined with DBS [47]. In the past decade, significant advancements have been made in creating mitochondria-targeted antioxidants (MTAs) by attaching antioxidants to the lipophilic cation triphenylphosphonium (TPP). Due to its positive charge and lipid solubility, TPP can readily cross biological membranes and accumulate within mitochondria, driven by their highly negative membrane potential. This results in up to a 500-fold increase in mitochondrial concentration, greatly enhancing antioxidant potency. Using this approach, Murphy and colleagues developed orally active MTAs such as MitoQ, MitoVitE and MitoTEMPOL, which offer superior protection against mitochondrial oxidative stress and hold therapeutic potential for neurodegenerative diseases, especially Parkinson’s disease [15].

#### 4.1.2. Control of Calcium Homeostasis and Excitotoxicity

Disruption of calcium homeostasis plays a key role in Parkinson’s disease, where α-synuclein aggregation and LRRK2 mutations promote Ca^2+^ dyshomeostasis, excitotoxicity, and dopaminergic neuronal death. Excitotoxic neuronal death is caused by abnormal glutamatergic function and intracellular calcium overload in PD [48]. Dopamine loss in PD leads to overactivity of the STN, causing glutamate-mediated excitotoxic damage to dopaminergic neurons in the substantia nigra. This creates a vicious cycle of neuronal loss, suggesting that reducing STN overactivity or blocking glutamate signaling could offer neuroprotection. Hyperactivity of the STN is reduced by DBS and decreases excitatory glutamatergic drive toward dopaminergic neurons improving the motor symptoms of PD. This decrease in excitability inhibits calcium-mediated activation of the apoptotic cascade and protects neuronal integrity [49].

Emerging evidence suggests that DBS may additionally influence excitotoxicity through more immediate biophysical modulation of calcium dynamics. High frequency stimulation can induce rapid membrane depolarization that transiently inactivates voltage gated calcium channels or alters their opening probability, thereby shaping calcium influx independent of long-term circuit changes. Astrocytes, which are abundant in basal ganglia output regions and intrinsically calcium active, also respond directly to DBS. Stimulation can evoke calcium release from intracellular stores via IP3 dependent pathways or modify store operated calcium entry, while stimulation induced shifts in the glutamate GABA balance affect astrocytic calcium transients. These glial responses influence gliotransmitter release including ATP, D serine, and glutamate, which in turn modulates neuronal excitability. Together, these neuron and astrocyte specific mechanisms indicate that DBS may stabilize calcium homeostasis through both circuit level normalization and rapid direct modulation of excitatory signaling [50].

#### 4.1.3. Inhibition of Apoptotic Signaling

Apoptosis and autophagy are essential for maintaining neuronal homeostasis, and their dysregulation contributes to dopaminergic neuron loss in Parkinson’s disease. Preclinical studies suggest that DBS may influence these pathways by reducing apoptotic signaling and promoting autophagic activity. STN DBS has been associated with decreased caspase 3 activation and an increased BCL2 to BAX ratio, findings that indicate reduced pro apoptotic drive and enhanced neuronal survival [51]. DBS related modulation of BCL2 has also been linked to increased autophagic clearance of toxic proteins, including alpha synuclein, in experimental models, although these effects have not been demonstrated in humans. Importantly, these changes appear to occur without major alterations in inflammatory markers or lipid peroxidation, suggesting a relatively specific effect on cell survival pathways rather than broad neurochemical changes. In addition, DBS induced increases in brain derived neurotrophic factor have been reported in some animal studies, supporting potential enhancement of neuronal resilience and synaptic maintenance [52]. Taken together, these findings indicate that DBS may engage molecular pathways relevant to cell survival; however, the evidence remains limited to preclinical models, and the translational significance of these mechanisms in patients with Parkinson’s disease remains uncertain.

#### 4.1.4. Synaptic Remodeling and Plasticity

STN-DBS affects neuronal firing and synaptic plasticity in both the STN and substantia nigra pars reticulata in a frequency-dependent manner. High-frequency DBS suppresses STN and SNr activity creates transient silent periods and modulates synaptic facilitation and depression, indicating that enhanced inhibitory synaptic plasticity may underlie its therapeutic effects. These results underscore the potential of optimizing DBS parameters, including closed-loop approaches, to maximize neurophysiological and clinical benefits in Parkinson’s disease [53]. In addition to neuroprotection, DBS promotes synaptic reorganization, increasing dendritic spine density, restoring long-term potentiation, and enhancing synaptic vesicle cycling. These structural and functional changes help stabilize network activity and support long-term cellular resilience [54].

### 4.2. Glial Contributions

#### 4.2.1. Astrocytic Modulation

Astrocytes play crucial roles in modulating neuronal survival and function in Parkinson’s disease. They can protect dopaminergic neurons by clearing glutamate from the extracellular space and releasing trophic factors and antioxidants [55]. DBS exerts its effects not only by directly modulating neurons but also by activating astrocytes, which release gliotransmitters, regulate synaptic activity, propagate signals across networks, and contribute to synaptic plasticity and neuroprotection. High-frequency stimulation, a key mode of DBS, directly activates astrocytes, leading to Ca^2+^ waves, ATP/adenosine release, and modulation of glutamate signaling, which together suppress pathological neuronal activity and tremor. These glial–neuronal interactions likely underlie the complex, network-wide and long-term effects of DBS [56]. In addition, DBS-activated astrocytes have been found to secrete neurotrophic molecules like BDNF and GDNF and contribute directly to the survival of dopaminergic neurons [57]. Astrocytes thus serve as a central hub through which DBS-driven electrical fields and neurotransmitter release translate into downstream circuit and molecular effects. STN stimulation increases glutamate and GABA release within the SNr, which directly activates astrocytic AMPA, mGlu, and GABA A receptors to generate robust Ca^2+^ elevations. These Ca^2+^ waves, recruited in a stimulation-dependent manner, trigger the release of gliotransmitters (ATP, glutamate, D-serine) and neurotrophic factors, enabling astrocytes to modulate local synaptic activity while also engaging pro-survival signaling pathways. In this way, astrocytic Ca^2+^ signaling provides a mechanistic bridge linking the immediate neurochemical effects of DBS to broader plasticity, metabolic support, and potential neuroprotective outcomes [50]. Conversely, under pathological conditions, astrocytes may contribute to neurodegeneration by releasing pro-inflammatory cytokines and other harmful molecules. These context-dependent roles highlight astrocytes as key players in PD pathogenesis and represent promising targets for novel therapeutic strategies [55].

#### 4.2.2. Microglial Polarization

Microglia are the brain’s resident immune cells with highly adaptable morphology and functions. Beyond their classical roles in immune defense, phagocytosis, and clearance of debris, microglia contribute to neuronal survival, differentiation, synaptic development, and plasticity. They release neurotrophic factors like IGF-1, BDNF, NGF, and FGF, support synaptic pruning and maturation, regulate dopaminergic axon wiring, and monitor synaptic function, highlighting their crucial homeostatic and developmental roles in the CNS [57]. Astrocytes and microglia interact to drive neuroinflammation, with microglia activating inflammatory pathways and producing ROS that trigger inflammasome activation. In turn, astrocytes respond to microglial signals (e.g., TNF-α), proliferate, release inflammatory mediators, and disrupt neuroglial metabolic coupling, collectively contributing to neuronal dysfunction [58]. DBS has been found to transform microglia from a pro-inflammatory M1 type to an anti-inflammatory, neuroprotective M2 type. It reduces cytokine secretion (e.g., TNF-α, IL-1β) and enhances anti-inflammatory mediators (e.g., IL-10, TGF-β). Through rebalancing of microglial activation state, DBS reduces chronic neuroinflammation and downstream toxicity [22,23].

#### 4.2.3. Preservation of Oligodendroglia

Oligodendrocytes play a key role in maintaining the myelin sheath and neural repair. Oligodendroglia also contributes to neuroprotection mediated by DBS. Experimental findings indicate that STAT5B, a transcription factor, is downregulated in PD oligodendrocytes due to DNMT3A-mediated promoter hypermethylation, leading to myelin deficits and impaired dopaminergic signaling. Restoring STAT5B expression rescues myelination and motor function, suggesting a potential therapeutic target and biomarker for PD [59].

### 4.3. Molecular Signaling Pathways

#### 4.3.1. Neurotrophic Factor Upregulation

One of the best-established molecular outcomes of DBS is the upregulation of neurotrophic factors. STN-DBS enhances the expression of BDNF, GDNF and VEGF in both experimental models and human tissue. These factors promote neuronal survival, synaptic plasticity, axonal regeneration, and angiogenesis, collectively supporting a microenvironment conducive to repair [60,61]. BDNF supports dopaminergic neuron integrity and correlates with disease severity; GDNF plays a pivotal role in maintaining midbrain dopaminergic function; VEGF promotes neurogenesis, synaptic plasticity, and neuroprotection [61]. BDNF–TrkB signaling appears to play a central role in mediating both the long-term neuroprotective effects and the acute motor benefits observed with STN-DBS [62]. However, despite encouraging preclinical results, clinical trials involving direct infusion of GDNF in PD patients have shown limited success, with reports of antibody formation and dose-dependent toxicity in non-human primates, raising safety concerns. Neurturin, a related trophic molecule delivered via viral vectors, demonstrated dopaminergic protection in animal models but yielded modest benefits in early human trials, warranting further investigation in larger studies. In addition, neuroimmunophilin ligands—proteins that promote neuronal growth and interact with immunosuppressive agents—have exhibited neuroprotective potential in PD models, although early clinical results have not confirmed efficacy. Overall, while DBS-induced trophic upregulation represents a promising avenue, translating these molecular effects into consistent clinical neuroprotection remains an ongoing challenge [12].

#### 4.3.2. Anti-Inflammatory and Antioxidant Signaling

In PD, α-synuclein accumulation activates microglia, triggering NF-κB signaling and the release of proinflammatory cytokines. T cells and gut dysbiosis further promote neuroinflammation, contributing to dopaminergic neuron degeneration. It simultaneously increases antioxidant protection by superoxide dismutase (SOD) and catalase upregulation and forestalls oxidative damage [62]. PD patients exhibit a persistent inflammatory profile, likely due to recruitment of activated monocytes, macrophages, and T lymphocytes to the CNS. DBS exerts therapeutic effects in part by modulating this inflammatory environment [63]. DBS may slow PD progression by reducing systemic inflammation, similar to the effects seen with levodopa therapy, potentially protecting neurons. However, the precise cellular and molecular mechanisms remain unclear, and further research is needed [64].

#### 4.3.3. Autophagy and Protein Homeostasis

PD pathology centers on abnormal α-synuclein aggregation. Key mechanisms include impaired protein clearance, mitochondrial dysfunction, and oxidative stress, which drive α-syn aggregation and neurodegeneration. Extracellular vesicles (EVs) have emerged as promising biomarkers since they carry α-synuclein and other molecular indicators from the brain into the blood and CSF. EV-associated α-syn plays dual roles—spreading pathology between neurons and aiding in clearance through glial uptake—and its altered levels in CSF and plasma may serve as sensitive biomarkers for PD progression and therapeutic monitoring [65]. Evidence suggests that DBS upregulates autophagic flux and proteasomal activity and enhances the clearance of misfolded proteins. Through normalization of proteostasis, DBS reduces aggregate burden and resulting cellular toxicity suggesting that they may have disease-modifying effects beyond symptom relief in neurodegenerative disorders [18,66].

#### 4.3.4. Epigenetic and Transcriptomic Modulation

Epigenetic mechanisms—such as DNA methylation, histone modification, and non-coding RNA expression—play key roles in Parkinson’s disease and its response to therapies. Emerging evidences indicate that DBS induces epigenetic modifications and transcriptomic changes in the blood of PD patients, reflecting neural responses such as enhanced cholinergic signaling and reduced neuroinflammation. Transcriptomic profiles of the activated brain regions reveal gene modifications of mitochondrial biogenesis, synaptic function, and immune regulation. DBS-targeted microRNAs (miRNAs) may also regulate protein synthesis of survival and stress handling and represent a new axis of molecular neuroprotection. Soreq et al. (2013) identified a specific panel of miRNAs whose altered expression normalized after DBS, suggesting that circulating leukocyte profiles could serve as accessible biomarkers for monitoring disease state and therapy response in PD [67,68]. Excessive histone deacetylation also contributes to PD pathology, suggesting that restoring epigenetic balance with histone deacetylase inhibitors (HDACIs) could also offer neuroprotective benefits [69].

#### 4.3.5. Systems-Level Integration: From Circuit to Cell

Pathological disruption of basal ganglia circuits in PD leads to overactive STN glutamatergic neurons, impaired thalamocortical signaling, and excitotoxicity, contributing to motor deficits. DBS alleviates PD symptoms by modulating abnormal basal ganglia activity and oscillatory patterns, but its mechanisms extend beyond local electrophysiological effects to include widespread neurotransmitter alterations across dopaminergic, glutamatergic, GABAergic, serotonergic, and cholinergic systems, potentially driving both therapeutic benefits and side effects [70]. DBS has evolved into a form of neuromodulation that integrates electrical and neurochemical approaches, enabling personalized therapy through improved understanding of disease mechanisms and biomarkers [71,72]. DBS also normalizes circuit-level activity across connected regions, including cortex and supplementary motor areas, effectively isolating pathological nodes and allowing the network to regain near-physiological function. Importantly, different stimulation frequencies (e.g., 60 Hz vs. 130 Hz) can differentially affect axial versus limb motor symptoms, illustrating the nuanced circuit-level effects of neuromodulation [73].

Neuroprotection caused by DBS is a multilevel system interaction of electrophysiological modulation promoting adaptive network and neuroprotective processes at the cellular and molecular level [8,74].

## 5. Translational and Therapeutic Implications

The recent understanding that deep brain stimulation (DBS) produces cellular and molecular neuroprotective mechanisms has important clinical application consequences for Parkinson’s disease (PD). Previously considered a therapy of end-stage motor symptoms, DBS may come to be envisioned as a potential disease-modifying treatment if these mechanisms are proven out in human studies [75]. This shift would require a greater holistic approach to patient selection, careful patient stratification, stimulation parameters, and outcome assessment [73]. One of the key translational prospects is the identification of biomarkers of neuroprotection induced by DBS. Biochemical markers of α-synuclein, neurofilament light chain, mediators of oxidative stress and inflammatory cytokines measured in cerebrospinal fluid or plasma can provide molecular indices of the effectiveness of DBS [38]. Neuroimaging tools such as PET and SPECT can potentially follow dopaminergic function, and high-field MRI can quantify cerebral blood flow and network integrity [37,76]. At the same time, local field potential and oscillation dynamic recordings by electrophysiology may provide real-time indexes of circuit modulation and molecular signaling. A combination of these multimodal biomarkers within longitudinal studies would allow more precise monitoring of neuroprotective end points [74].

Invasive neuromodulation, particularly DBS, has evolved from a reversible lesioning tool to a sophisticated interface for modulating dysfunctional neural circuits, with preclinical studies playing a key role in refining mechanisms, optimizing efficacy, and translating findings to improve treatment of PD and other movement disorders [77]. Standard continuous high-frequency DBS is effective at treating symptoms, but new data are prompting alternative stimulation patterns that preferentially activate neuroprotective mechanisms [78]. Also explored are stimulation of other targets besides the subthalamic nucleus such as globus pallidus internus or pedunculopontine nucleus that may have unique mechanistic advantages toward neuroprotection [52]. A combination of DBS with other disease-modifying strategies is yet another promising area. Co-administration of neurotrophic factor, gene therapy, drug therapies directed against mitochondrial dysfunction or protein aggregation, and indeed cell-based transplantation therapies can complement DBS and augment protective mechanisms. By these mechanisms, DBS can potentially serve as a platform technology for multimodal neuroprotective therapies [79].

Adaptive or closed-loop DBS devices that can adjust stimulation based on the output of physiology may better maintain useful signaling and diminish side effects. Closed-loop DBS uses real-time neural signals to adapt stimulation automatically, improving PD outcomes and potentially expanding DBS applications to psychiatric disorders as well [80]. Closed-loop DBS in PD simulates suppression of beta-band activity in the thalamo-cortico-basal ganglia loop, achieving better symptom control and lower power consumption than open-loop DBS [81,82]. Adaptive DBS uses real-time biomarkers from multi-channel microelectrodes to adjust stimulation and reduce PD symptoms. Non-invasive and experimental approaches, such as low-frequency fields or optogenetic stimuli, aim to target deep neurons without surgery, though these remain largely experimental in animal models [82].

Among the cellular mechanisms implicated in PD pathogenesis, calcium dysregulation, oxidative stress, mitochondrial dysfunction, impaired autophagy, and abnormal α-synuclein accumulation and clearance are all potential targets for neuroprotective therapies, several of which are currently under investigation in clinical trials [83]. Early DBS can reduce both the dosage and complexity of Parkinson’s disease medications while providing long-term motor benefits compared with standard medical therapy [84]. Earlier DBS intervention offers the potential to improve quality of life and functional ability in Parkinson’s disease patients, providing significant symptomatic relief over a longer period [85]. Clinical trials have demonstrated that DBS can influence glucose metabolism, gray matter volume, and the connectivity and activity of brain regions involved in memory and cognition. Ongoing multicenter trials are expected to provide further evidence on whether DBS can produce a clinically meaningful slowing of disease progression and establish it as a recognized therapeutic option, placing it alongside emerging disease-modifying therapies [86].

Emerging evidence suggests that different neuromodulatory interventions in PD—including DBS, low-intensity focused ultrasound (LIFUS), and stereotactic radiosurgical techniques such as Gamma Knife—may share overlapping mechanisms that contribute to neuroprotection. STN-DBS has been shown to restore pathological network dynamics by suppressing excessive beta oscillations (13–35 Hz) and enhancing narrowband gamma activity within the cortico-basal ganglia circuitry, thereby improving motor control and possibly inducing downstream molecular effects through altered neuronal firing and metabolic activity. Similarly, LIFUS exerts non-thermal mechanical effects on neuronal membranes, activating mechanosensitive channels such as TRPA1 and Piezo, which lead to calcium influx, astrocytic glutamate release, and NMDA receptor activation—mechanisms that promote neurotrophic signaling and enhance GDNF and BDNF expression. In contrast, Gamma Knife and MR-guided focused ultrasound (MRgFUS) lesions achieve functional modulation through targeted ablation, which disrupts pathological circuit synchrony while potentially triggering secondary plasticity and neuroinflammatory repair pathways. Together, these techniques appear to converge on a common outcome—restoring network balance and promoting trophic and synaptic resilience—highlighting that both invasive and non-invasive modalities may induce neuroprotective responses via modulation of oscillatory activity and glia-neuron signaling [87,88].

Overall, translating mechanistic understanding into clinical practice involves the determination of strong biomarkers, optimization of stimulation approaches, merging with adjunct therapies, and patient stratification using biological signatures [73]. Despite the challenges, progress in PD research—such as gene-based animal models, novel clinical trial designs, and adaptive analytic approaches—offers hope for developing effective neuroprotective therapies in the near future [89].

## 6. Limitations

The current evidences on DBS-mediated neuroprotection is constrained by several important limitations that warrant careful interpretation. First, most human studies are underpowered, relying on small, often non-randomized cohorts with substantial variability in disease duration, medication status, and DBS parameters. Such heterogeneity limits the ability to detect true neuroprotective effects and increases susceptibility to false-positive or parameter-dependent findings. Second, follow-up periods are generally short, and most outcomes depend on clinical measures of motor performance that are highly sensitive to symptomatic benefit; thus, apparent “neuroprotection” may reflect stimulation-induced functional compensation rather than structural preservation. Third, mechanistic conclusions are frequently extrapolated from preclinical models that only partially mimic human PD, particularly toxin models that produce rapid dopaminergic loss but lack chronic α-synuclein aggregation, multisystem involvement, or progressive inflammatory changes. Genetic or α-synuclein seeding models improve fidelity but still fail to replicate the full temporal and regional complexity of human disease.

Fourth, there is substantial inconsistency in molecular endpoints, with different studies measuring neurotrophic factors, inflammatory markers, mitochondrial function, or synaptic proteins at varying time points and in different brain regions. This lack of standardized biomarkers hampers cross-study comparability and obscures whether reported molecular changes are sustained, causal, or merely epiphenomena. Fifth, publication bias and selective reporting are underrecognized limitations. Positive mechanistic findings (e.g., increased BDNF, reduced apoptosis, enhanced autophagy) are more frequently published than null results, which may distort the perceived coherence of the field. Moreover, most mechanistic studies sample tissue only after survival to the experimental endpoint, creating a form of survivor bias that may overrepresent neurons already resilient to disease or stimulation. Finally, the intrinsic heterogeneity of PD and atypical parkinsonian syndromes—driven by genetic factors, molecular phenotypes, and clinical subtypes—suggests that DBS-related neuroprotection is unlikely to generalize uniformly across patients. These limitations collectively highlight the need for longitudinal, biomarker-integrated, multi-center studies, as well as more sophisticated preclinical models that simultaneously capture molecular, circuit-level, and behavioral outcomes.

## 7. Conclusions and Future Directions

Deep brain stimulation remains one of the most effective symptomatic treatments for Parkinson’s disease, reliably improving motor function and quality of life. Preclinical studies across toxin-based, genetic, and α-synuclein models consistently show that STN-DBS can engage cellular and molecular pathways associated with neuroprotection—including enhanced neurotrophic signaling, reduced oxidative and inflammatory stress, modulation of calcium dynamics, strengthened mitochondrial resilience, and improved synaptic and glial function. These findings provide a strong mechanistic foundation for the hypothesis that DBS may influence disease biology.

However, in humans, the evidence for true neuroprotection remains inconclusive. Clinical improvements under stimulation, divergence of imaging signals, and perioperative biomarker changes are best interpreted as physiological or compensatory effects occurring during active stimulation, not as proof of slowed neurodegeneration. No existing human study has demonstrated that DBS modifies long-term progression independent of its symptomatic action. Thus, while the biological rationale is increasingly compelling, DBS cannot yet be considered a disease-modifying therapy for Parkinson’s disease. What this review demonstrates is not that DBS does slow PD progression, but that DBS engages multiple molecular and cellular pathways that could, in principle, support neuroprotection if appropriately harnessed or combined with complementary therapies. Moving forward, rigorous longitudinal studies incorporating standardized biomarkers, improved imaging, adaptive stimulation strategies, disease-model-aligned animal work, and mechanistically targeted combinatorial trials will be essential to determine whether DBS can translate its preclinical neuroprotective signatures into clinically meaningful disease modification. In summary, DBS is a powerful neuromodulatory therapy with promising mechanistic features that may support neuroprotection, but definitive disease-modifying effects in humans remain unproven. Continued translational research is needed to bridge this gap. Future research should prioritize large, longitudinal, biomarker-guided studies that directly test whether DBS slows neurodegeneration rather than simply improving symptoms. Combining molecular biomarkers with next-generation imaging and electrophysiological readouts will help clarify when and in whom protective effects occur. There is also a need to standardize biomarker panels and outcome measures so that results across studies can be meaningfully compared. Refining stimulation strategies—such as adaptive, phase-specific, or patterned DBS—may enhance pro-survival signaling while reducing stimulation burden. More advanced preclinical models that incorporate chronic α-synuclein pathology, neuroinflammation, and network dysfunction are essential for understanding mechanism. Combinatorial approaches, including pairing DBS with gene therapy, neurotrophic factors, or disease-modifying drugs, may uncover synergistic benefits that are not seen with DBS alone. Ultimately, integrating genetic background, molecular signatures, and network physiology into patient selection could support a more individualized approach, allowing DBS to be tailored to specific disease subtypes and biological profiles.

DBS now stands at a pivotal point in its evolution. Although it remains a symptomatic therapy, emerging mechanistic insights raise the possibility that, with targeted innovation and rigorous study, DBS could eventually be developed into an intervention that modifies disease biology. At the intersection of mechanistic insight and translational innovation, future research has the potential not only to deepen our understanding of PD pathophysiology but also to fundamentally alter the disease trajectory for patients.

## Data Availability

No new data were created or analyzed in this study. Data sharing is not applicable to this article.

## References

[1] Eser P., Kocabicak E., Bekar A., Temel Y. (2024). Insights into neuroinflammatory mechanisms of deep brain stimulation in Parkinson’s disease. Exp. Neurol..

[2] McKinnon C., Gros P., Lee D.J., Hamani C., Lozano A.M., Kalia L.V., Kalia S.K. (2018). Deep brain stimulation: Potential for neuroprotection. Ann. Clin. Transl. Neurol..

[3] Stefani A., Cerroni R., Mazzone P., Liguori C., Di Giovanni G., Pierantozzi M., Galati S. (2019). Mechanisms of action underlying the efficacy of deep brain stimulation of the subthalamic nucleus in Parkinson’s disease: Central role of disease severity. Eur. J. Neurosci..

[4] Xu W., Wang J., Li X.N., Liang J., Song L., Wu Y., Liu Z., Sun B., Li W.G. (2023). Neuronal and synaptic adaptations underlying the benefits of deep brain stimulation for Parkinson’s disease. Transl. Neurodegener..

[5] Nordi T.M., Gounella R.H., Luppe M., Junior J.N.S., Fonoff E.T., Colombari E., Romero M.A., Carmo J.P.P.D. (2022). Low-Noise Amplifier for Deep-Brain Stimulation (DBS). Electronics.

[6] Benabid A.L. (2003). Deep brain stimulation for Parkinson’s disease. Curr. Opin. Neurobiol..

[7] Ashkan K., Rogers P., Bergman H., Ughratdar I. (2017). Insights into the mechanisms of deep brain stimulation. Nat. Rev. Neurol..

[8] Jakobs M., Fomenko A., Lozano A.M., Kiening K.L. (2019). Cellular, molecular, and clinical mechanisms of action of deep brain stimulation-a systematic review on established indications and outlook on future developments. EMBO Mol. Med..

[9] Bucur M., Papagno C. (2023). Deep Brain Stimulation in Parkinson Disease: A Meta-analysis of the Long-term Neuropsychological Outcomes. Neuropsychol. Rev..

[10] Tuominen R.K., Renko J.M. (2024). Biomarkers of Parkinson’s disease in perspective of early diagnosis and translation of neurotrophic therapies. Basic Clin. Pharmacol. Toxicol..

[11] Lang A.E., Espay A.J. (2018). Disease Modification in Parkinson’s Disease: Current Approaches, Challenges, and Future Considerations. Mov. Disord..

[12] Sarkar S., Raymick J., Imam S. (2016). Neuroprotective and Therapeutic Strategies against Parkinson’s Disease: Recent Perspectives. Int. J. Mol. Sci..

[13] Alster P., Otto-Ślusarczyk D., Szlufik S., Duszyńska-Wąs K., Drzewińska A., Wiercińska-Drapało A., Struga M., Kutyłowski M., Friedman A., Madetko-Alster N. (2024). The significance of glial cell line-derived neurotrophic factor analysis in Progressive Supranuclear Palsy. Sci. Rep..

[14] Ammal Kaidery N., Tarannum S., Thomas B. (2013). Epigenetic landscape of Parkinson’s disease: Emerging role in disease mechanisms and therapeutic modalities. Neurotherapeutics.

[15] Jin H., Kanthasamy A., Ghosh A., Anantharam V., Kalyanaraman B., Kanthasamy A.G. (2014). Mitochondria-targeted antioxidants for treatment of Parkinson’s disease: Preclinical and clinical outcomes. Biochim. Biophys. Acta.

[16] Gogna T., Housden B.E., Houldsworth A. (2024). Exploring the Role of Reactive Oxygen Species in the Pathogenesis and Pathophysiology of Alzheimer’s and Parkinson’s Disease and the Efficacy of Antioxidant Treatment. Antioxidants.

[17] Dorszewska J., Kowalska M., Prendecki M., Piekut T., Kozłowska J., Kozubski W. (2021). Oxidative stress factors in Parkinson’s disease. Neural Regen. Res..

[18] Lee E.J., Aguirre-Padilla D.H., Fomenko A., Pawar G., Kapadia M., George J., Lozano A.M., Hamani C., Kalia L.V., Kalia S.K. (2024). Reduction of alpha-synuclein oligomers in preclinical models of Parkinson’s disease by electrical stimulation in vitro and deep brain stimulation in vivo. Brain Stimul..

[19] Breydo L., Wu J.W., Uversky V.N. (2012). A-synuclein misfolding and Parkinson’s disease. Biochim. Biophys. Acta.

[20] Varma D., Sen D. (2015). Role of the unfolded protein response in the pathogenesis of Parkinson’s disease. Acta Neurobiol. Exp..

[21] Wang E.S., Yao H.B., Chen Y.H., Wang G., Gao W.W., Sun Y.R., Guo J.G., Hu J.W., Jiang C.C., Hu J. (2013). Proteomic analysis of the cerebrospinal fluid of Parkinson’s disease patients pre- and post-deep brain stimulation. Cell. Physiol. Biochem..

[22] Araújo B., Caridade-Silva R., Soares-Guedes C., Martins-Macedo J., Gomes E.D., Monteiro S., Teixeira F.G. (2022). Neuroinflammation and Parkinson’s Disease-From Neurodegeneration to Therapeutic Opportunities. Cells.

[23] Collins L.M., Toulouse A., Connor T.J., Nolan Y.M. (2012). Contributions of central and systemic inflammation to the pathophysiology of Parkinson’s disease. Neuropharmacology.

[24] Bellucci A., Mercuri N.B., Venneri A., Faustini G., Longhena F., Pizzi M., Missale C., Spano P. (2016). Review: Parkinson’s disease: From synaptic loss to connectome dysfunction. Neuropathol. Appl. Neurobiol..

[25] Calabresi P., Mechelli A., Natale G., Volpicelli-Daley L., Di Lazzaro G., Ghiglieri V. (2023). Alpha-synuclein in Parkinson’s disease and other synucleinopathies: From overt neurodegeneration back to early synaptic dysfunction. Cell Death Dis..

[26] Toader C., Tataru C.P., Munteanu O., Serban M., Covache-Busuioc R.A., Ciurea A.V., Enyedi M. (2024). Decoding Neurodegeneration: A Review of Molecular Mechanisms and Therapeutic Advances in Alzheimer’s, Parkinson’s, and ALS. Int. J. Mol. Sci..

[27] Chen Y., Zhu G., Yuan T., Ma R., Zhang X., Meng F., Yang A., Du T., Zhang J. (2024). Subthalamic nucleus deep brain stimulation alleviates oxidative stress via mitophagy in Parkinson’s disease. NPJ Park. Dis..

[28] Benabid A.L., Piallat B., Wallace B., Benazzouz A., Lenartz D., Andressen C., Krack P., Pollak P. (2003). Might Deep Brain Stimulation of the Subthalamic Nucleus Be Neuroprotective in Patients with Parkinson’s Disease?. Thalamus Relat. Syst..

[29] Pal G., Ouyang B., Verhagen L., Serrano G., Shill H.A., Adler C.H., Beach T.G., Kordower J.H. (2018). AZSAND Probing the striatal dopamine system for a putative neuroprotective effect of deep brain stimulation in Parkinson’s disease. Mov. Disord..

[30] Wu S.T., Ma Y., Zhang K., Zhang J.G. (2012). Effect of deep brain stimulation on substantia nigra neurons in a rat model of Parkinson’s disease. Chin. Med. J..

[31] Musacchio T., Rebenstorff M., Fluri F., Brotchie J.M., Volkmann J., Koprich J.B., Ip C.W. (2017). Subthalamic nucleus deep brain stimulation is neuroprotective in the A53T α-synuclein Parkinson’s disease rat model. Ann. Neurol..

[32] Huang C., Chu H., Ma Y., Zhou Z., Dai C., Huang X., Fang L., Ao Q., Huang D. (2019). The neuroprotective effect of deep brain stimulation at nucleus basalis of Meynert in transgenic mice with Alzheimer’s disease. Brain Stimul..

[33] Wichmann T., Bergman H., DeLong M.R. (2018). Basal ganglia, movement disorders and deep brain stimulation: Advances made through non-human primate research. J. Neural Transm..

[34] Wallace B.A., Ashkan K., Heise C.E., Foote K.D., Torres N., Mitrofanis J., Benabid A.L. (2007). Survival of midbrain dopaminergic cells after lesion or deep brain stimulation of the subthalamic nucleus in MPTP-treated monkeys. Brain.

[35] Vitek J.L., Johnson L.A. (2019). Understanding Parkinson’s disease and deep brain stimulation: Role of monkey models. Proc. Natl. Acad. Sci. USA.

[36] Stoof J.C., Winogrodzka A., van Muiswinkel F.L., Wolters E.C., Voorn P., Groenewegen H.J., Booij J., Drukarch B. (1999). Leads for the development of neuroprotective treatment in Parkinson’s disease and brain imaging methods for estimating treatment efficacy. Eur. J. Pharmacol..

[37] Paschali A., Constantoyannis C., Angelatou F., Vassilakos P. (2013). Perfusion brain SPECT in assessing motor improvement after deep brain stimulation in Parkinson’s disease. Acta Neurochir..

[38] Mugge L., Krafcik B., Pontasch G., Alnemari A., Neimat J., Gaudin D. (2019). A Review of Biomarkers Use in Parkinson with Deep Brain Stimulation: A Successful Past Promising a Bright Future. World Neurosurg..

[39] Sanmartino F., Cano-Cano F., Rashid-López R., Cruz-Gómez Á.J., Lozano-Soto E., Macías-García P., Sánchez-Fernández F.L., López-Sosa F., Gómez-Jaramillo L., Riqué-Dormido J. (2024). Significance of neurodegeneration and neuroplasticity serum biomarkers in Parkinson’s disease patients treated with subthalamic stimulation. NPJ Park. Dis..

[40] Mallach A., Weinert M., Arthur J., Gveric D., Tierney T.S., Alavian K.N. (2019). Post mortem examination of Parkinson’s disease brains suggests decline in mitochondrial biomass, reversed by deep brain stimulation of subthalamic nucleus. FASEB J..

[41] deSouza R.M., Moro E., Lang A.E., Schapira A.H. (2013). Timing of deep brain stimulation in Parkinson disease: A need for reappraisal?. Ann. Neurol..

[42] de la Fuente-Fernández R., Schulzer M., Mak E., Sossi V. (2010). Trials of neuroprotective therapies for Parkinson’s disease: Problems and limitations. Park. Relat. Disord..

[43] Devos D., Hirsch E., Wyse R. (2021). Seven Solutions for Neuroprotection in Parkinson’s Disease. Mov. Disord..

[44] Pelletier S.J., Cicchetti F. (2014). Cellular and molecular mechanisms of action of transcranial direct current stimulation: Evidence from in vitro and in vivo models. Int. J. Neuropsychopharmacol..

[45] Exner N., Lutz A.K., Haass C., Winklhofer K.F. (2012). Mitochondrial dysfunction in Parkinson’s disease: Molecular mechanisms and pathophysiological consequences. EMBO J..

[46] Guidetti M., Bertini A., Pirone F., Sala G., Signorelli P., Ferrarese C., Priori A., Bocci T. (2022). Neuroprotection and Non-Invasive Brain Stimulation: Facts or Fiction?. Int. J. Mol. Sci..

[47] Gao X.Y., Yang T., Gu Y., Sun X.H. (2022). Mitochondrial Dysfunction in Parkinson’s Disease: From Mechanistic Insights to Therapy. Front. Aging Neurosci..

[48] Nam D., Kim H., Han S.J., Son I., Ho D.H. (2024). Effects of Calcium Ion Dyshomeostasis and Calcium Ion-Induced Excitotoxicity in Parkinson’s Disease. J. Mol. Pathol..

[49] Rodriguez M.C., Obeso J.A., Olanow C.W. (1998). Subthalamic nucleus-mediated excitotoxicity in Parkinson’s disease: A target for neuroprotection. Ann. Neurol..

[50] Barat E., Boisseau S., Bouyssières C., Appaix F., Savasta M., Albrieux M. (2012). Subthalamic nucleus electrical stimulation modulates calcium activity of nigral astrocytes. PLoS ONE.

[51] Liu J., Liu W., Yang H. (2019). Balancing Apoptosis and Autophagy for Parkinson’s Disease Therapy: Targeting BCL-2. ACS Chem. Neurosci..

[52] Rajneesh C.P., Hsieh T.H., Chen S.C., Lai C.H., Yang L.Y., Chin H.Y., Peng C.W. (2020). Deep Brain Stimulation of the Pedunculopontine Tegmental Nucleus Renders Neuroprotection through the Suppression of Hippocampal Apoptosis: An Experimental Animal Study. Brain Sci..

[53] Milosevic L., Kalia S.K., Hodaie M., Lozano A.M., Fasano A., Popovic M.R., Hutchison W.D. (2018). Neuronal inhibition and synaptic plasticity of basal ganglia neurons in Parkinson’s disease. Brain.

[54] Jahan I., Harun-Ur-Rashid M., Islam M.A., Sharmin F., Al Jaouni S.K., Kaki A.M., Selim S. (2026). Neuronal plasticity and its role in Alzheimer’s disease and Parkinson’s disease. Neural Regen. Res..

[55] Rappold P.M., Tieu K. (2010). Astrocytes and therapeutics for Parkinson’s disease. Neurotherapeutics.

[56] Fenoy A.J., Goetz L., Chabardès S., Xia Y. (2014). Deep brain stimulation: Are astrocytes a key driver behind the scene?. CNS Neurosci. Ther..

[57] Domingues A.V., Pereira I.M., Vilaça-Faria H., Salgado A.J., Rodrigues A.J., Teixeira F.G. (2020). Glial cells in Parkinson’s disease: Protective or deleterious?. Cell. Mol. Life Sci..

[58] Meng H., Wei J.H., Yu P.Z., Ren J.X., Tang M.Y., Sun J.Y., Yan X.Y., Su J. (2023). Insights into Advanced Neurological Dysfunction Mechanisms Following DBS Surgery in Parkinson’s Patients: Neuroinflammation and Pyroptosis. Curr. Issues Mol. Biol..

[59] Li Y., Su Z., Zhai J., Liu Q., Wang H., Hao J., Tian X., Gao J., Geng D., Wang L. (2025). Oligodendrocyte-Specific STAT5B Overexpression Ameliorates Myelin Impairment in Experimental Models of Parkinson’s Disease. Cells.

[60] Rangasamy S.B., Soderstrom K., Bakay R.A., Kordower J.H. (2010). Neurotrophic factor therapy for Parkinson’s disease. Prog. Brain Res..

[61] Faust K., Vajkoczy P., Xi B., Harnack D. (2021). The Effects of Deep Brain Stimulation of the Subthalamic Nucleus on Vascular Endothelial Growth Factor, Brain-Derived Neurotrophic Factor, and Glial Cell Line-Derived Neurotrophic Factor in a Rat Model of Parkinson’s Disease. Stereotact. Funct. Neurosurg..

[62] Fischer D.L., Kemp C.J., Cole-Strauss A., Polinski N.K., Paumier K.L., Lipton J.W., Steece-Collier K., Collier T.J., Buhlinger D.J., Sortwell C.E. (2017). Subthalamic Nucleus Deep Brain Stimulation Employs trkB Signaling for Neuroprotection and Functional Restoration. J. Neurosci..

[63] Dong-Chen X., Yong C., Yang X., Chen-Yu S., Li-Hua P. (2023). Signaling pathways in Parkinson’s disease: Molecular mechanisms and therapeutic interventions. Signal Transduct. Target. Ther..

[64] Chang L., Dong W.W., Luo B., Qiu C., Lu Y., Lin X.J., Zhang W.B. (2023). Deep brain stimulation improves central nervous system inflammation in Parkinson’s disease: Evidence and perspectives. CNS Neurosci. Ther..

[65] Picca A., Guerra F., Calvani R., Romano R., Coelho-Júnior H.J., Bucci C., Marzetti E. (2021). Mitochondrial Dysfunction, Protein Misfolding and Neuroinflammation in Parkinson’s Disease: Roads to Biomarker Discovery. Biomolecules.

[66] Pei H., Wu Z., Ma L., Wang J., Li J., Geng X., Zou Y., Zhang M., Qi R., Yu H. (2024). Deep Brain Stimulation Mechanisms in Parkinson’s Disease: Immediate and Long-Term Effects. J. Integr. Neurosci..

[67] Consales C., Merla C., Marino C., Benassi B. (2018). The epigenetic component of the brain response to electromagnetic stimulation in Parkinson’s Disease patients: A literature overview. Bioelectromagnetics.

[68] Song N., Du J., Gao Y., Yang S. (2020). Epitranscriptome of the ventral tegmental area in a deep brain-stimulated chronic unpredictable mild stress mouse model. Transl. Neurosci..

[69] Harrison I.F., Dexter D.T. (2013). Epigenetic targeting of histone deacetylase: Therapeutic potential in Parkinson’s disease?. Pharmacol. Ther..

[70] Alosaimi F., Boonstra J.T., Tan S., Temel Y., Jahanshahi A. (2022). The role of neurotransmitter systems in mediating deep brain stimulation effects in Parkinson’s disease. Front. Neurosci..

[71] Kim S., Kang S., Kim J., Lee D., Kim S., Lee J., Jang K.I., Oh Y.S., Rah J.C., Huh M.S. (2021). Closed-Loop Neuromodulation for Parkinson’s Disease: Current State and Future Directions. IEEE Trans. Mol. Biol. Multi-Scale Commun..

[72] Wolf D., Ayon-Olivas M., Sendtner M. (2024). BDNF-Regulated Modulation of Striatal Circuits and Implications for Parkinson’s Disease and Dystonia. Biomedicines.

[73] Davidson B., Milosevic L., Kondrataviciute L., Kalia L.V., Kalia S.K. (2024). Neuroscience fundamentals relevant to neuromodulation: Neurobiology of deep brain stimulation in Parkinson’s disease. Neurotherapeutics.

[74] Oyovwi M.O., Babawale K.H., Jeroh E., Ben-Azu B. (2025). Exploring the role of neuromodulation in neurodegenerative disorders: Insights from Alzheimer’s and Parkinson’s diseases. Brain Disord..

[75] Harnack D., Kupsch A. (2010). The impact of subthalamic deep brain stimulation on nigral neuroprotection-myth or reality?. Neuromodulation.

[76] Lokkegaard A., Werdelin L.M., Regeur L., Karlsborg M., Jensen S.R., Brødsgaard E., Madsen F.F., Lonsdale M.N., Friberg L. (2007). Dopamine transporter imaging and the effects of deep brain stimulation in patients with Parkinson’s disease. Eur. J. Nucl. Med. Mol. Imaging.

[77] Chen J., Volkmann J., Ip C.W. (2024). A framework for translational therapy development in deep brain stimulation. NPJ Park. Dis..

[78] Dong J., Cui Y., Li S., Le W. (2016). Current Pharmaceutical Treatments and Alternative Therapies of Parkinson’s Disease. Curr. Neuropharmacol..

[79] Cummings J. (2017). Disease modification and Neuroprotection in neurodegenerative disorders. Transl. Neurodegener..

[80] Bouthour W., Mégevand P., Donoghue J., Lüscher C., Birbaumer N., Krack P. (2019). Biomarkers for closed-loop deep brain stimulation in Parkinson disease and beyond. Nat. Rev. Neurol..

[81] Fleming J.E., Dunn E., Lowery M.M. (2020). Simulation of Closed-Loop Deep Brain Stimulation Control Schemes for Suppression of Pathological Beta Oscillations in Parkinson’s Disease. Front. Neurosci..

[82] Rama Raju V., Anji Reddy D., Narsimha D., Srinivas K., Kavitha Rani B. (2021). Adaptive Closed-Loop Deep Brain Stimulator Coding Techniques for Target Detections in Parkinson’s. IETE J. Res..

[83] Athauda D., Foltynie T. (2015). The ongoing pursuit of neuroprotective therapies in Parkinson disease. Nat. Rev. Neurol..

[84] Hacker M.L., Turchan M., Heusinkveld L.E., Currie A.D., Millan S.H., Molinari A.L., Konrad P.E., Davis T.L., Phibbs F.T., Hedera P. (2020). Deep brain stimulation in early-stage Parkinson disease: Five-year outcomes. Neurology.

[85] Suarez-Cedeno G., Suescun J., Schiess M.C. (2017). Earlier Intervention with Deep Brain Stimulation for Parkinson’s Disease. Park. Dis..

[86] Jakobs M., Lee D.J., Lozano A.M. (2020). Modifying the progression of Alzheimer’s and Parkinson’s disease with deep brain stimulation. Neuropharmacology.

[87] Lee K.S., Clennell B., Steward T.G.J., Gialeli A., Cordero-Llana O., Whitcomb D.J. (2022). Focused Ultrasound Stimulation as a Neuromodulatory Tool for Parkinson’s Disease: A Scoping Review. Brain Sci..

[88] Ding H., Groppa S., Muthuraman M. (2022). Toward future adaptive deep brain stimulation for Parkinson’s disease: The novel biomarker—Narrowband gamma oscillation. Neural Regen. Res..

[89] Stocchi F., Olanow C.W. (2013). Obstacles to the development of a neuroprotective therapy for Parkinson’s disease. Mov. Disord..

